# Variations of Antioxidant Properties and NO Scavenging Abilities during Fermentation of Tea

**DOI:** 10.3390/ijms12074574

**Published:** 2011-07-15

**Authors:** Yang Xu, Hang Zhao, Min Zhang, Chun-Jie Li, Xue-Zhen Lin, Jun Sheng, Wei Shi

**Affiliations:** 1 Key Laboratory for Molecular Enzymology and Engineering of the Ministry of Education, College of Life Science, Jilin University, Changchun 130012, China; E-Mails: xuyang759@yahoo.com.cn (Y.X.); zhzky@163.com (H.Z.); zhangmin2584652@yahoo.com.cn (M.Z.); lichunjie0821@163.com (C.-J.L.); a-xing_3@163.com (X.-Z.L.); 2 Yunnan Research Centre for Advance Tea Processing, Yunnan Agricultural University, Kunming 650201, China

**Keywords:** tea, catechins, theabrownins, ROS, NO

## Abstract

Tea is known as one of the most popular beverages in the world, which is believed to be beneficial for health. The main components in tea will change a lot depending on the different processes of fermentation, and thus the effects of different teas on human health may differ. The aim of this study is to explore the varied abilities of reactive oxygen species (ROS) and nitric oxide (NO) scavenging during the fermentation of tea. In this study, we conducted the *in vitro* experiments which involved some reaction systems indicating the abilities of scavenging ROS and NO. We also investigated the effects of tea and their components (catechins, theabrownins, caffeine) on the intracellular levels of ROS and NO, using Raw 264.7 cells as the model. We found that regardless of whether it was out of cell system or in Raw 264.7 cells, the abilities of scavenging ROS would decrease during the fermentation of tea. Further, the post-fermented pu-erh tea showed the best effect on inhibiting the lipopolysaccharide (LPS)-induced production of NO. These findings indicated that the fermentation process caused a change of the components which might be due to the changes of their antioxidant properties and NO scavenging abilities.

## 1. Introduction

Tea is one of the most popular beverages. Tea consumption is part of many people’s daily life, as an everyday drink and as a therapeutic aid in many illnesses. Thus, it is worthy to be investigated and has attracted a lot of attention recently [1–3]. Tea is categorized into different types based on manufacturing process: unfermented tea (green tea), fully fermented tea (black tea) and post-fermented tea (pu-erh tea) [4,5]. In recent years, the legendary medicinal properties are well established. These beneficial effects have been attributed to the presence of tea components such as catechins, polysaccharides, theabrownins and caffeine [6–9]. The chemical compositions of the tea in different forms were distinct and induced the change of the bioactivities, depending on the degree of fermentation and the various modes of preparation [10].

**Figure 1:**
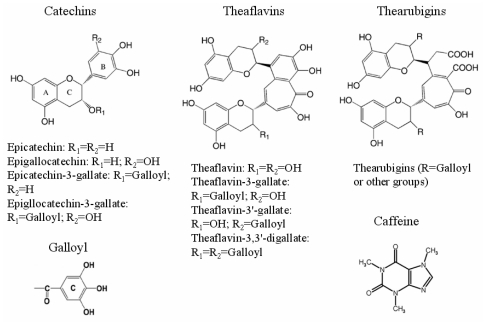
Chemical structures of the investigated components in tea (Adapted from [16]).

Catechins are a group of natural polyphenols found in green tea. There are four main catechin derivatives, including (−)-epicatechin, (−)-epigallocatechin, (−)-epicatechin gallate and (−)-epigallocatechin gallate. In the manufacture of black tea, the monomeric catechins undergo polyphenol oxidase-dependent oxidative polymerization leading to the formation of bisflavanols, theaflavins, thearubigins, and other oligomers in a process commonly known as “fermentation”. The major theaflavins in black tea are theaflavin, theaflavin-3-gallate, theaflavin-3′-gallate and theaflavin-3,3′-digallate. Theaflavins undergo further oxidation during the fermentation of black tea and pu-erh tea to form more polymerized thearubigins, and then condensed theabrownins [11–14]. The caffeine (1,3,7-trimethylxanthine) is a purine alkaloid found in more than 60 different plant species. Its amount in the tea varies little during the fermentation [15]. The chemical structures of components mentioned above are illustrated in Figure 1.

Reactive oxygen species (ROS) are generated as byproducts of normal aerobic metabolism and are increased under oxidative stress conditions, including superoxide anion, hydrogen peroxide, hydroxyl radical [17]. Because of their high reactivity, free radicals can damage diverse cellular macromolecules, including proteins, carbohydrates, lipids, and nucleic acids. Free radical-caused damage to these molecules has been implicated in the causation of some degenerative diseases. For example, destructive effects on proteins may play a role in cataract formation, oxidative damage to DNA may be involved in the development of certain cancers, and lipid oxidative damage can lead to the occurrence and progression of vascular disease [18]. Nitric oxide (NO) has a role in mediating macrophage cytotoxicity, regulating blood pressure, and neurotransmission. A well-balanced level of NO has shown to be an important regulator of various physiological processes, such as vasodilation, neurotransmission and host defense [19]. Once NO is formed in the cell, it can react with superoxide anions to form peroxynitrite, which is a potent oxidizing and nitrating molecule. Excess NO production by inducible nitric oxide synthase (iNOS) has been implicated in the development of several diseases such as cancer, diabetes, renal disease and cardiovascular disease [20]. Hence, a considerable number of investigations have been focused on the prevention of oxidative damage initiated by free radicals and damage by NO. Recently many researches have reported that tea have excellent ability of ROS and NO scavenging [21], but the changes of antioxidant properties and NO scavenging abilities during the fermentation of tea still remain unclear. Especially, the exact components which cause these variations and the way they affect the scavenging abilities of tea during the fermentation have not been reported so far.

In this study, our aim is to explore the changes of valid components during fermentation and their effects on the scavenging abilities of tea by choosing some representative teas with different degrees of fermentation. We used green tea, black tea, pu-erh tea and their main components to explore variations of antioxidant properties and NO scavenging abilities during fermentation of tea.

## 2. Results

### 2.1. Contents of Several Polyphenol Ingredients in Tea

In a previous study, we have already analyzed the contents of polyphenol ingredients in three kinds of tea [22]. The chemical components in green tea, black tea and pu-erh tea aqueous extracts were analyzed and compared. The polysaccharide levels were substantially higher in the fermented black tea and pu-erh tea, while the polyphenol level was higher in the unfermented green tea (Table 1).

### 2.2. DPPH Radical Scavenging Activity and ABTS^+^ Decolourisation Assay

**Figure 2:**
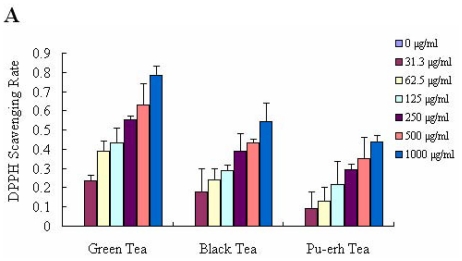

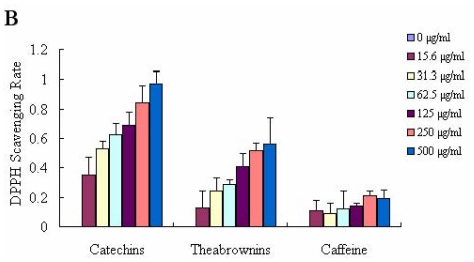
The abilities of DPPH scavenging: (**A**) DPPH scavenging rate of green tea, black tea and pu-erh tea extracts at the concentrations of 0–1000 μg/mL; (**B**) DPPH scavenging rate of catechins, theabrownins and caffeine at the concentrations of 0–500 μg/mL. The results were expressed as the mean of three independent experiments.

The method of 1,1-diphenyl-2-picrylhydrazyl radical (DPPH) scavenging assay is based on the reduction of DPPH, a stable free radical. Because of the odd electron of DPPH, it gives a strong absorption maximum at 517 nm by visible spectroscopy. As the odd electron of the radical becomes paired off in the presence of a hydrogen donor, that is, a free-radical scavenging antioxidant, the absorption strength is decreased, and the resulting decolorization is stoichiometric with respect to the number of electrons captured. This reaction has been widely used to test the ability of components to act as free-radical scavengers or hydrogen donors and to evaluate the antioxidative activity of foods and plant extracts [23]. Various concentrations of tea extracts (0–1000 μg/mL) and their main components (0–500 μg/mL) were incubated with DPPH for 30 min individually, and then their absorbance values at 517 nm was measured. The results presented the decrease in absorbance of the DPPH radical due to the scavenging abilities of the extracts of different teas (Figure 2A). The effects of the different tea components to scavenge DPPH radical increased with higher concentrations of tea extract, with an exception of caffeine (Figure 2B). The radical scavenging abilities of the tea extracts decreased in the following order during fermentation: green tea > black tea > pu-erh tea; catechins > theabrownins. The DPPH scavenging rate of green tea was 78.41% at the concentration of 1000 μg/mL, while the DPPH scavenging rate of pu-erh tea was only 44.12%. The similar antioxidant activity of tea and their components was also observed by 2,2′-azinobis-(3-ethylbenzothiazoline-6-sulfonicacid) radical cation (ABTS^+^) decolourisation assay. The results showed that inhibition of ABTS^+^ by different teas was consistent with that of DPPH (Table 2), and the tea components exhibited the similar inhibiting abilities of ABTS^+^ to that of DPPH (Table 3). Among all the three kinds of tea, green tea showed the best ability of scavenging ABTS^+^ (88.23% at 1000 μg/mL), and accordingly, catechins (90.23% at 500 μg/mL) inhibited ABTS^+^ more than theabrownins (50.99% at 500 μg/mL). This is mainly because of the high content of polyphenols in green tea, especially catechins.

### 2.3. NO Scavenging Effect

**Figure 3:**
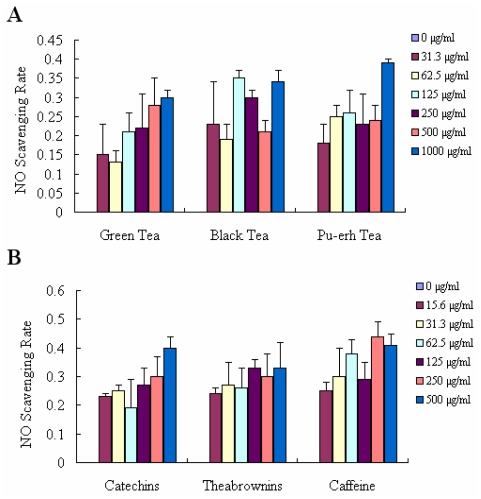
The abilities of NO scavenging. (**A**) NO scavenging rate of green tea, black tea and pu-erh tea extracts at concentrations of 0–1000 μg/mL; (**B**) NO scavenging rate of catechins, theabrownins and caffeine at concentrations of 0–500 μg/mL. The results were expressed as the mean of three independent experiments.

Nitric oxide (NO) reacts with superoxide (O_2_^−^) to form the peroxynitrite anion, which is a potential strong oxidant as the decomposition of this molecule produces hydroxyl radical and nitrogen dioxide. During recent years, it has become increasingly apparent that NO contributes significantly to oxidative cell damage. Therefore, it might be beneficial for human health if consumed foods could scavenge NO. As shown in Figure 3A, all of the three kinds of tea inhibited the NO production in non-cellular experiment to a smaller extent, but not in a dose-dependent manner. Furthermore they exhibited similar scavenging NO ability, with an individual value of 30.03%, 34.25% and 39.91% at a concentration of 1000 μg/mL. Similarly, the components in tea showed the same effects on inhibition of NO production, with an individual scavenging rate of 40.19%, 33.25% and 41.56% at 1000 μg/mL (Figure 3B). Therefore, the effects of scavenging NO may be less relevant at different degrees of tea fermentation.

### 2.4. Effect on Ameliorating H_2_O_2_-Induced Loss of Cell Viability and Decreasing H_2_O_2_-Induced Accumulation of ROS in Raw 264.7 Cells

**Figure 4:**
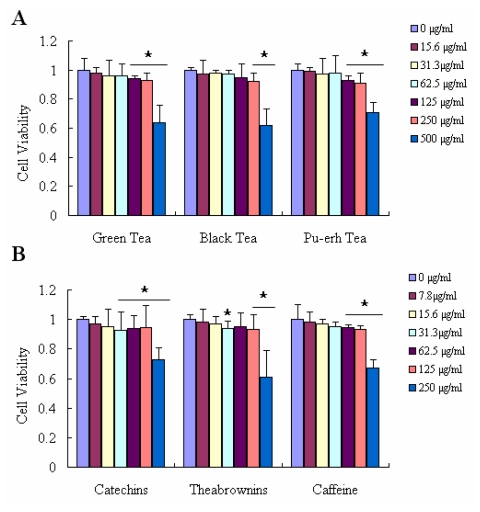
The viability of Raw 264.7 cells exposed to various components by MTT assay. (**A**) The cells were treated with three kinds of tea respectively at indicated doses for 24 h. Cell viability was determined by MTT assay; (**B**) The cells were treated with distinct tea components respectively at indicated doses for 24 h. The results were expressed as percentage of control and were represented by mean ± SE determined from three independent experiments. All *p* values were for comparisons between control and tea treated cells. The asterisk indicated *p* < 0.05 *versus* control.

**Figure 5:**
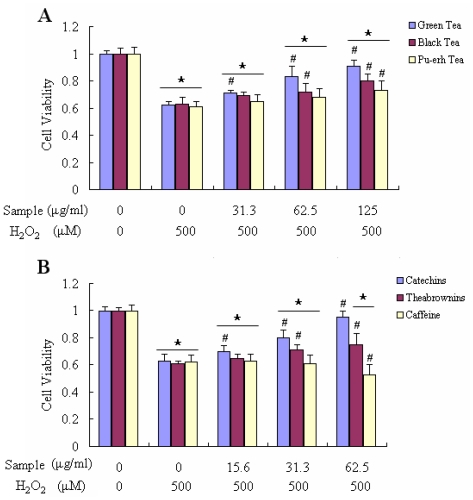
Protective effects of tea and their components on H_2_O_2_-induced decrease of Raw 264.7 cell viability. (**A**) Cell viability was estimated by MTT assay after treatment with H_2_O_2_ (500 μM) and together with one of the three tea extracts for 24 h (at indicated concentrations); (**B**) Cell viability was estimated by MTT assay after treatment with H_2_O_2_ (500 μM) and together with one of three tea’s main components for 24 h (at indicated concentrations). The results were expressed as percentage of control and were represented by mean ± SE determined from three independent experiments. All *p* values were for comparisons between control and tea treated cells. The asterisk indicated *p* < 0.05 *versus* control. # indicated *p* < 0.05 *versus* negative control.

To examine whether these tea extracts produce toxic effects on Raw 264.7 cells, we performed the cell viability assay. As shown in Figure 4A and B, in three teas (0–250 μg/mL) and their main components (0–125 μg/mL) treatment did not significantly affect cell viability. The data indicated that these tea extracts were relatively safe for Raw 264.7 cells at the above concentrations. Therefore, 31.3 μg/mL, 62.5 μg/mL, 125 μg/mL of three tea extracts and 15.6 μg/mL, 31.3 μg/mL, 62.5 μg/mL of their main components were used in the following experiments. The cell viability was expressed as 3-(4,5-dimethylthiazol-2-yl)-2,5-diphenyl tetrazolium bromide (MTT) conversion rate. The effect of three different fermentative tea extracts and their main components on H_2_O_2_-induced loss of Raw 264.7 cell viability as depicted in Figure 5. Treatment with 500 μM H_2_O_2_ for 24 h decreased the viability of Raw 264.7 cells about 40% relative to the negative control. After the cells were treated with H_2_O_2_, simultaneously co-treated with one of three teas at different concentrations (31.3–125 μg/mL) individually for 24 h, the cell viability was almost dose-dependent. Green tea showed the best protective effect on the damaged Raw 264.7 cells. Figure 5A shows that the cell ability reached up to 90.98% when the cells were co-incubated with 125 μg/mL green tea. The cell viability after treatment with catechins gradually increased in accordance with the concentration, which was more significant than that treated with theabrownins (Figure 5B). In accordance with DPPH and ABTS^+^ assay results, caffeine had no obvious effects on anti OH^−^ damage to cells either.

**Figure 6:**
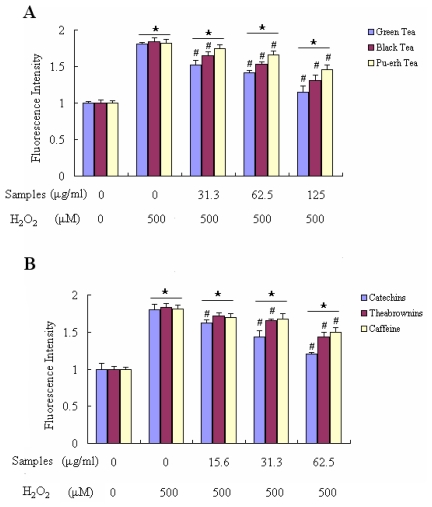
Tea and their components reduced H_2_O_2_-induced accumulation of ROS in Raw 264.7 cells. (**A**) The fluorescence intensity of DCF-DA was measured after Raw 264.7 cells were exposed to one of the three tea extracts and together to H_2_O_2_ (500 μM) for 24 h; (**B**) The fluorescence intensity of DCF-DA was measured after Raw 264.7 cells were exposed to one of the three tea components at indicated concentrations and together to H_2_O_2_ (500 μM) for 24 h. The results were expressed as percentage of control and were represented by mean ± SE determined from three independent experiments. All *p* values were for comparisons between control and tea treated cells. The asterisk indicated *p* < 0.05 *versus* control. # indicated *p* < 0.05 *versus* negative control.

ROS are the main factor that causes oxidative stress, which results in decreasing cell viability. The level of 2′,7′-dichlorofluorescin diacetate (DCF-DA) fluorescence is an indicator of ROS production. After treatment with 500 μM H_2_O_2_ for 24 h, the DCF-DA fluorescence intensity increased about 70–80% in comparison with the negative control. In this experiment, the cells were treated with 500 μM H_2_O_2_, and were simultaneously co-incubated with one of the three teas or one of their three main components at the indicated concentrations. As shown in Figure 6 A and B, the increase in the DCF-DA fluorescence intensity was eliminated partly when the cells were treated with different concentrations of the three teas (31.3–125 μg/mL) and their main components (15.6–62.5 μg/mL). At the concentration of 125 μg/mL, green tea showed the best inhibition of ROS formation in cells among the three teas, which decreased 66.21% fluorescence intensity more than the control group (Figure 6A). As to their main components in different degrees of fermented tea, the less oxidized catechins had better effect on scavenging intracellular free radical than more polymerized theabrownins. Figure 6 B showed that 62.5 μg/mL catechins decreased the fluorescence intensity by 59.48%, while theabrownins at the same concentration decreased only 38.93% fluorescence intensity.

### 2.5. Suppressing Effects of Tea Extracts on LPS-Induced NO Production in Macrophages

**Figure 7:**
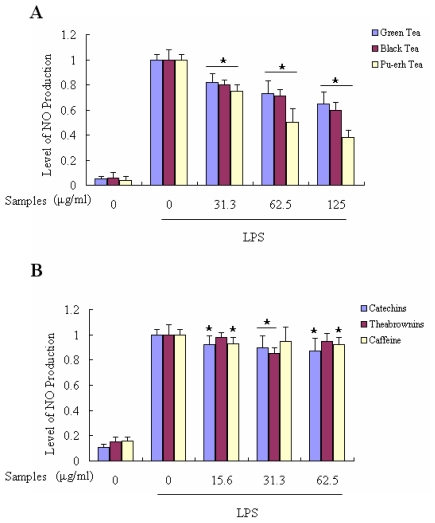
Effect of teas with various fermentation degrees on the NO production in LPS-activated Raw264.7 cells. (**A**) The level of NO was measured after Raw 264.7 cells were exposed to one of the three tea extracts and LPS (1 μg/mL) for 24 h; (**B**) The level of NO was measured after Raw 264.7 cells were exposed to one of the three tea components at indicated concentrations and LPS (1 μg/mL) for 24 h. The results were expressed as percentage of control and were represented by mean ± SE determined from three independent experiments. All *p* values were for comparisons between control and tea treated cells. The asterisk indicated *p* < 0.05 *versus* negtive control.

The Raw 264.7 cells were treated with 1 μg/mL lipopolysaccharide (LPS) to induce NO production. LPS-treated cells produced a high level of nitrite which was about 10~20-fold higher than that produced by the unstimulated cells. Control cells were only treated with 1 μg/mL LPS. Significant concentration-dependent inhibition of NO production was observed when cells were co-treated with LPS and various concentrations of tea extracts or their components for 24 h. Among the three teas, pu-erh tea had the most visible effects in restraining the cells to generate NO in a dose-dependent way (Figure 7A). When Raw 264.7 cells were incubated with 125 μg/mL pu-erh tea extract, the cells could only produce 38.18% NO compared to the control group, while the level of NO production was 65.00% with 125 μg/mL green tea. However, the main components, catechins, theabrownins and caffeine, did not decrease NO production of LPS-induced Raw 264.7 cells very much, as shown in Figure 7B.

## 3. Experimental Section

### 3.1. Materials

Dulbecco’s modified Eagle medium (DMEM), newborn calf serum, and 3-(4,5-dimethylthiazol-2-yl)-2,5-diphenyltetrazoliunbromide (MTT) were purchased from GIBCO BRL (Grand Island, NY, USA). Trypsin, penicillin, streptomycin, DPPH, ABTS, DCF-DA and all other chemicals employed in this study were of analytical grade and were purchased from Sigma Chemical Co (St. Louis, USA). Catechins, theabrownins and caffeine were provided by China Academy of Pu-erh Tea Research (Pu Erh, Yunnan, China). The tea fermentation processes are carried out by tea-making experts in the China Academy of Pu-erh Tea Research. The resulting tea products are classified according to the degree of fermentation as unfermented tea (green tea), fully fermented tea (black tea) and post-fermented tea (pu-erh tea), and they were provided by China Academy of Pu-erh Tea Research (Pu Erh, Yunnan, China).

### 3.2. Preparation of Tea Extracts [22]

The tea leaves of Green tea, Black tea and Pu-erh tea were collected from plants grown in the Yunnan Highlands of China. Green tea leaves were collected and heated, dried at <60 °C and molded to make unfermented tea. To make fermented Black tea and post fermented pu-erh tea, the tea leaves were dampened and fermented. Then dried at <60 °C and packed. Green tea, Black tea and Pu-erh tea were extracted three times by placing in boiling distilled water for 10 min each time. The solution was collected, lyophilized to obtain the aqueous extract.

### 3.3. Determination of Polyphenol, Polysaccharides, and Caffeine Content in Concentrated Tea Extracts [22]

Polyphenol content were performed under the guidelines of national standards using the ferrous tartrate method. Briefly, the tea extraction solution, buffer solution and ferrous tartrate tetrahydrate solution were mixed in 25-mL capacity bottle. Absorbance (A) at 540 nm with a 10 mm quartz cell was used to calculate the extraction of tea polyphenols. Polysaccharides were quantitated using the anthrone–sulfuric acid method using glucose as standard as described. A standard curve was generated with glucan, which was linear between the concentration range of 5 and 30 μg. The calibration curve equation was y = 0.063× + 0.0579 and had a correlation coefficient of R^2^ = 0.9957. Caffeine was quantitated using the lead subacetate method. A standard curve was generated with caffeine, which was linear between the concentration range of 50 and 300 μg. The calibration curve equation was y = 62.911× + 0.0058 and had a correlation coefficient of R^2^ = 0.9997.

### 3.4. DPPH-Radical Scavenging Activity

The DPPH method [24] was employed here to evaluate the free radical scavenging abilities of various samples. Tea extract samples were dissolved in distilled methanol, and solutions of different concentrations were prepared in different test tubes. 4 mL of 0.1 mM DPPH solution in methanol was added to these test tubes and shaken vigorously. The tubes were then incubated in the dark at room temperature for 30 min. A DPPH blank sample was prepared without any extract, and methanol was used for the baseline correction. Changes (or decrease) in the absorbance at 517 nm were measured using a UV visible spectrophotometer (SHIMADZU UV 2550). The DPPH solution was freshly prepared daily, stored in a flask covered with aluminum foil, and kept in the dark at 4 °C between measurements. The percent decrease in the absorbance was recorded for each concentration, and percent quenching of DPPH was calculated on the basis of the observed decrease in absorbance of the radical. The radical scavenging activity was expressed as the inhibition percentage and was calculated using the following formula:

$$\%\text{DPPH scavenging}=[({\text{A}}_{\text{control}}-{\text{A}}_{\text{sample}})\times 100/{\text{A}}_{\text{control}}]$$

### 3.5. ABTS^+^ Decolorization Assay

ABTS^+^ radical scavenging activities of the samples were determined by the method of Re *et al.* [25]. In brief, ABTS^+^ was dissolved in water to a 7 mM concentration. ABTS^+^ radical cation was produced by reacting ABTS^+^ stock solution with 2.45 mM potassium persulfate (final concentration) and allowing the mixture to stand in the dark at room temperature for 16 hours before use. For the determination of antioxidant activity, the ABTS^+^ radical solution was diluted with ethanol to an absorbance of 0.70 (±0.02) at 734 nm. After addition of 1.0 mL of diluted ABTS^+^ radical solution to 10 mL of the sample, the absorbance was recorded in 5 minutes after initial mixing. Percentage inhibition was calculated by using the following equation:

$$\%{\text{ABTS}}^{+}\;\text{scavenging}=[({\text{A}}_{\text{control}}-{\text{A}}_{\text{sample}})\times 100/{\text{A}}_{\text{control}}]$$

### 3.6. NO-Scavenging Activity

To estimate possible NO-scavenging activity of tea extracts, different concentrations of sodium nitroprusside (SNP) (1, 2 or 5 mM) were incubated alone or in combination with different concentrations of tea extracts. All solutions were made in 0.1 M phosphate buffer (pH 7.4) and added directly into tubes. SNP was an inorganic complex where NO was found as NO^+^ and light irradiation was necessary for release of NO. Therefore, incubation mixtures were incubated on light, at room temperature, and nitrite levels were determined exactly after 30 min. For nitrite measurements, sample aliquots of 1 mL were mixed with an equal volume of Greiss reagent system [26] containing 1% sulphanilamide and 0.1% naphthylethylenediamine in 5% phosphoric acid. The mixture was then incubated at room temperature for 10 min and the absorbance was measured at 546 nm [27].

### 3.7. Cell Culture and Assessment of Cell Viability

Raw 264.7 cells were cultured in DMED supplemented with 10% ednotoxin-free, heat-inactivated fetal calf serum (Gibco, Grand Island, NY). Cell viability was measured by quantitative colorimetric assay with MTT, showing the mitochondrial activity of living cells as described in the literature [28,29]. Cells were cultured at a density of 2 × 10^5^ cells/mL on 96-well plates and cultured 24 h before treatment. Initially, the culture medium was replaced with fresh medium containing various concentrations of tea extracts or components, to determine the optimal concentration of tea or components for the following experiments (Figure 4). In the following experiments (Figure 5), tea extracts were added together with H_2_O_2_ for 24 h. After co-treatment with H_2_O_2_ and tea for 24 h, the medium was removed and fresh medium containing 0.5 mg/mL MTT was added to each well, followed by incubation for 3 h at 37 °C. Finally the medium containing MTT was removed, and cells were lysed with dimethyl sulfoxide (DMSO). The absorbance at 570 nm was measured on a Bio-Rad 3350 microplate reader. Control cells were treated in the same way without H_2_O_2_, and the value of different absorbance was expressed as a percentage of control.

### 3.8. Measurement of Intracellular Reactive Oxygen Species

The level of intracellular reactive species was quantified by fluorescence with DCF-DA as described by Yamamoto *et al.* [30]. DCF-DA is a cell permeable and nonfluorescent agent. Cells (2 × 10^5^ cells/well) were incubated with Hallam’s physiological saline (HPS) containing DCF-DA (10 μM) in a 96-well microplate for 30 min at 37 °C. After the incubation, cells were washed three times with HPS and incubated with HPS containing samples (with different concentration) for 30 min, then incubated with HPS containing H_2_O_2_ (500 μM) for the indicated time periods. The intracellular reactive species levels were measured by using a fluorescence plate reader (Fluoroskan Ascent 2.4), at an excitation wavelength of 485 nm and an emission wavelength of 538 nm. The measured fluorescence values were expressed as a percentage of the fluorescence in control cells.

### 3.9. Determination of NO Production

To determine the effect of tea extract on the NO production, the cells were seeded at a density of 2 × 10^5^ cells per well in 96-well culture dishes. Following incubation for 24 h, the adherent cells were washed three times with Phosphate Buffered Saline (PBS). The cells were then incubated in the medium with extracts from one of three teas or components, with or without 1 μg/mL LPS. After incubation for 24 h, the medium was collected and stored at −70 °C until assay. Finally, medium nitrite concentration was measured as an indicator of NO production by the Griess reaction [26]. Control cells were only treated with LPS without tea, and the value of different levels of NO production was expressed as a percentage of control.

### 3.10. Statistical Analysis

The results were presented as mean ± SE. Student’s t test was used for a statistical comparison. (A probability level of 5%, *p* < 0.05 was considered statistically significant).

## 4. Discussion

Our previous study has shown that the degree of fermentation had a profound effect on the levels of polyphenols. Total polyphenol levels in tea aqueous were significantly decreased during fermentation, which were the most beneficial components for human health. In addition, polysaccharide levels increased during the fermentation process. The caffeine content of different teas did not change much with the degree of fermentation [22]. Thus, the distinct physiologic functions of tea will vary during fermentation. The aim of this study is to explore the changes of antioxidant properties and NO scavenging abilities during fermentation of tea.

Free-radical-initiated autoxidation of cellular membrane lipids could lead to cellular necrosis and a variety of pathological conditions such as cancer and even aging [37]. Hence, a considerable number of investigations have focused on the prevention of oxidative damage initiated by free radicals. In this study, we found that green tea exhibited the strongest ability of scavenging ROS, such as DPPH and ABTS^+^. As fermentation went on, the effect of antioxidation would attenuate, which coincided with the result that the catechins’ capacity of decreasing extracellular and intracellular ROS was better than theabrownins’. Catechins are a group of natural polyphenols most enriched in green tea, which is known as a powerful deoxidizer due to its antioxidative active group, such as phenolic hydroxyl group. When the tea undertgoes fermentation, catechins would react with each other to generate polymers, known as theaflavins. Then theaflavins continue to converge into thearubigins. After post fermentation, the small molecular polyphenols polymerize into polymer with higher molecular weight, theabrownins. Throughout the entire reaction course, the antioxidative active groups are lost due to polymerization. Thus, the reduction of ability to clearing ROS may result from the fact that the polyphenols and their effective antioxidative activity would abate during fermentation.

NO is a gaseous free radical that can be formed by nitric oxide synthase (NOS) in cells. Following induction, iNOS can be expressed quantitatively in various cells, such as macrophages, smooth muscle cells and hepatocytes. However, if the balance of NO *in vivo* is disrupted, a lot of damage would be caused to the body, leading to conditions such as cancer, diabetes, renal disease and cardiovascular disease [39–41]. One aim of our study is to find out whether the function of tea to inhibit NO production would vary under different degrees of fermentation. Interestingly, opposite to the effects on scavenging the oxidative free radicals, the post-fermentated pu-erh tea displayed a better function on NO suppression according to Figure 7A. This result may have little relevance with the variations of the polyphenols in tea, according to the effect on inhibition of NO production as shown in Figure 7B. The results in Figure 3 show that in the non-cellular experiment, the effects of three tea extracts and their components on scavenging NO did not increase in a dose-dependent way, while in the cellular experiment (Figure 7) three teas inhibited the cell to produce NO in a dose-dependent manner but for their components. The reason may be that the active ingredients of the tea can permeate into or be absorbed by the cells, and suppress the expressions or the functions of NOS or other related proteins. That the pu-erh tea showed the most effective inhibition on NO generation was probably caused by the increase of polysaccharide levels during the fermentation process.

The results in this study will provide us clues to find new drugs against diseases caused by overproduction of ROS or NO.

## 5. Conclusions

The results acquired from chemical components and scavenging activity analysis in this study provide clues to the key molecules that contribute to the inhibition of ROS and NO production. The data showed that catechins, the main green tea phenolic components, had the best ability of scavenging ROS and helping cells to survive against H_2_O_2_ damage, which indicated that the antioxidative effects of tea would attenuate during fermentation. However, the abilities of tea to inhibit NO production in cells were elevated when tea was fermented, rather than the components in tea not directly scavenging NO outside the cells. Hence, we proposed that the components of tea might permeate into or be absorbed by cells, and then they function to restrain the proteins which were in charge of NO production. Although further studies are required to elucidate the molecular mechanism, these results demonstrated that tea extract and their constituents could be candidate agents for the therapy of diseases caused by overoxidation or NO.

## Figures and Tables

**Table 1:** Contents of several polyphenol ingredients in teas [22]^a^.

Sample (%w/w)	Polyphenols	Polysaccharides	Caffeine	Theabrownins
Green Tea	56.23 ± 5.17^b^	1.01 ± 0.11^b^	8.62 ± 0.14^b^	–
Black Tea	42.40 ± 3.35^c^	3.42 ± 0.05^c^	8.92 ± 0.19^b^	–
Pu-erh Tea	33.13 ± 3.18^d^	4.81 ± 0.13^d^	9.31 ± 0.09^b^	7.32–10.50

a Data are expressed as mean ± SD of n = 3;

b–d values in the same column followed by a different letter represent a significant difference at p < 0.05.

**Table 2:** ABTS^+^ scavenging rate of three tea extracts at different concentrations^a^.

Sample	Concentration (μg/mL)						
	0	31.3	62.5	125	250	500	1000
Green Tea	0	30.21 ± 0.83^b^	40.75 ± 0.23^c^	50.47 ± 0.76^d^	62.49 ± 0.58^e^	78.22 ± 0.12^f^	88.23 ± 0.73^g^
Black Tea	0	22.38 ± 0.62^b^	25.22 ± 0.21^c^	30.37 ± 0.28^d^	39.10 ± 0.62^e^	45.21 ± 0.13^f^	58.12 ± 0.91^g^
Pu-erh Tea	0	11.35 ± 0.91^b^	20.01 ± 0.12^c^	25.46 ± 0.84^d^	30.23 ± 0.62^e^	31.2 ± 0.94d,e	45.00 ± 0.61^f^

a Data are expressed as mean ± SD of n = 3;

b–g values in the same line followed by a different letter represent a significant difference at p < 0.05.

**Table 3:** ABTS^+^ scavenging rate of three tea components at different concentrations^a^.

Sample	Concentration (μg/mL)						
	0	15.6	31.3	62.5	125	250	500
Catechins	0	22.12 ± 0.42^b^	35.40 ± 0.80^c^	42.23 ± 0.71^d^	63.54 ± 0.38^e^	77.33 ± 0.13^f^	90.23 ± 0.71^g^
Theabrownins	0	13.32 ± 0.92^b^	25.34 ± 0.23^c^	29.13 ± 0.21^d^	33.59 ± 0.22^e^	39.98 ± 0.41^f^	50.99 ± 0.62^g^
Caffeine	0	9.09 ± 0.72^b^	12.78 ± 0.72^c^	7.29 ± 0.51^d^	11.32 ± 0.32^e^	15.45 ± 0.72^f^	19.54 ± 0.97^g^

a Data are expressed as mean ± SD of n = 3;

b–g values in the same line followed by a different letter represent a significant difference at p < 0.05.
